# Solar Disinfection of *Pseudomonas aeruginosa* in Harvested Rainwater: A Step towards Potability of Rainwater

**DOI:** 10.1371/journal.pone.0090743

**Published:** 2014-03-03

**Authors:** Muhammad T. Amin, Mohsin Nawaz, Muhammad N. Amin, Mooyoung Han

**Affiliations:** 1 Alamoudi Water Research Chair, King Saud University, Riyadh, Kingdom of Saudi Arabia; 2 Department of Civil and Environmental Engineering, College of Engineering, King Faisal University, Al-Hofuf Al-Ahsa, Kingdom of Saudi Arabia; 3 Civil and Environmental Engineering Department, Seoul National University, Shinrimdong, KwanakGu, Seoul, South Korea; University of Missouri-Kansas City, United States of America

## Abstract

Efficiency of solar based disinfection of *Pseudomonas aeruginosa (P. aeruginosa)* in rooftop harvested rainwater was evaluated aiming the potability of rainwater. The rainwater samples were exposed to direct sunlight for about 8–9 hours and the effects of water temperature (°C), sunlight irradiance (W/m^2^), different rear surfaces of polyethylene terephthalate bottles, variable microbial concentrations, pH and turbidity were observed on *P. aeruginosa* inactivation at different weathers. In simple solar disinfection (SODIS), the complete inactivation of *P. aeruginosa* was obtained only under sunny weather conditions (>50°C and >700 W/m^2^) with absorptive rear surface. Solar collector disinfection (SOCODIS) system, used to improve the efficiency of simple SODIS under mild and weak weather, completely inactivated the *P. aeruginosa* by enhancing the disinfection efficiency of about 20% only at mild weather. Both SODIS and SOCODIS systems, however, were found inefficient at weak weather. Different initial concentrations of *P. aeruginosa* and/or *Escherichia coli* had little effects on the disinfection efficiency except for the SODIS with highest initial concentrations. The inactivation of *P. aeruginosa* increased by about 10–15% by lowering the initial pH values from 10 to 3. A high initial turbidity, adjusted by adding kaolin, adversely affected the efficiency of both systems and a decrease, about 15–25%; in inactivation of *P. aeruginosa* was observed. The kinetics of this study was investigated by Geeraerd Model for highlighting the best disinfection system based on reaction rate constant. The unique detailed investigation of *P. aeruginosa* disinfection with sunlight based disinfection systems under different weather conditions and variable parameters will help researchers to understand and further improve the newly invented SOCODIS system.

## Introduction

Water shortage and adequate cost-effective water treatment technologies are the major concerns of this era around the globe. According to the latest available information by the joint study of UNICEF and WHO, still 783 million people do not have access to safe water resources [1]. The use of polluted water for ingestion, especially in many areas of developing countries, is the major cause of diarrhea and various other water-borne diseases. Over 1.2 million children, most being less than five years old, die due to diarrheal diseases each year [2]. Currently the alternate water sources and efficient water treatment technologies are needed. Rainwater harvesting (RWH) as safe water source and cost-effective solar disinfection (SODIS) system has significant importance in this regard [3]–[5]. For efficient utilization of harvested rainwater, the RWH system should consist of its catchment area, a storage tank, a treatment facility, a supply facility and pipes [6]. Roof being an impermeable catchment area is a good source to collect rainwater. However the microbial quality of harvested rainwater can be deteriorated due to the possible contamination upon contact with catchment which is one of the major constraints of using rainwater for potable purposes [7], [8]. *P. aeruginosa* is a part of microbial contamination which is considered responsible for nosocomial infection in immune-compromised people [9]. The presence of this microorganism is unacceptable as it has been implicated in waterborne and food borne diseases and is now considered primary infectious agent [10]. *P. aeruginosa* is detected in stored rainwater by many researchers which is of serious health concerns for water users [11]–[15].

SODIS is being used as point-of use treatment method in many parts of the world. It is believed that solar energy can be used in improving water quality in those parts of the world that experience hot, sunny climates [16], [17]. The portion of solar radiation which plays a major role in generating the antimicrobial activity is believed to be UV-A, 315–400nm, and to a lesser extent visible violet and blue light in the range of 400–490nm [18], [19]. In addition, the increase in water temperature through solar collector is known to work synergistically with solar radiation to further enhance the efficiency of SODIS process [20], [21]. The inactivation of high populations of microorganisms in highly turbid water (approximately 200 nephelometric turbidity units, NTU) can be achieved within 7h if the water temperature reached at least 55°C [22]. Previous studies found that children, between the ages of 5–16 years, who stored their drinking water in 1.5 liters polyethylene terephthalate (PET) bottles in direct sunlight were less prone to diarrhea and other water borne diseases compared to the children who kept their water bottles out of the sun [23], [24]. The efficiency of SODIS and solar collector disinfection (SOCODIS) systems was tested against total and fecal coliform, *Escherichia coli* (*E. coli*) and HPC previously [4] while the efficiency of SODIS and SOCODIS systems was checked against the inactivation of *P. aeruginosa* in this study.

The current study focuses on harvested rainwater as a potential potable water source by using cost-effective solar disinfection of *P. aeruginosa*. The objectives include the enhancement of *P. aeruginosa* inactivation using different rear surface of PET bottles and to investigate the efficiency of solar collector under mild and weak weather conditions with different concentrations of the tested organism. The effects of different initial pH and turbidity values (adjusted by adding kaolin) on *P. aeruginosa* inactivation are also the important part of this study in addition to investigating the effects of the presence of *E. coli* in different concentrations.

## Materials and Methods

### Sampling sites and disinfection setup

The rainwater storage tank was installed in one of the educational buildings (engineering building #39) at the Campus of Seoul National University, South Korea. The harvested rainwater from the rooftop of the building was used mainly for toilet flushing inside building. Rainwater samples were collected in 8 sterile PET bottles of 2L capacity from the supply point which is situated at an height of 1.35 m from the base of the underground rainwater storage tank. The PET bottles were filled ¾ full and were shaken for about 20 seconds to oxygenate the water and an air space of about 15% of the bottle volume was still kept for aeration [16].

**Figure 1:**
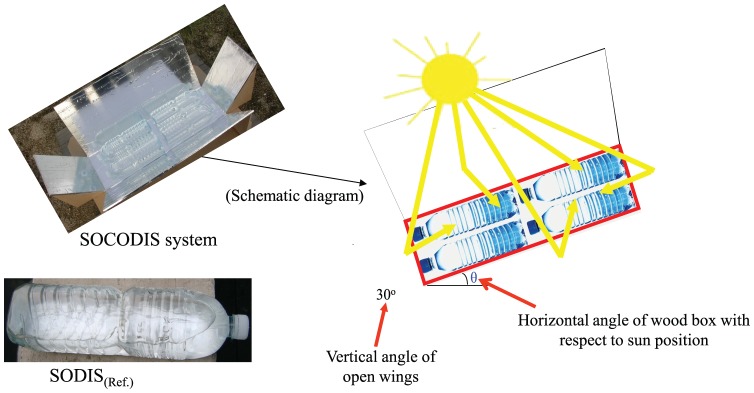
Demonstration of SODIS_(Ref.)_ and SOCODIS systems.

In simple SODIS, 3 PET bottles with transparent, absorptive (rear surface painted black) and reflective (rear surface covered with aluminium foil) rear surfaces were exposed to natural sunlight while 4 transparent PET bottles kept inside the box were exposed to sunlight at the same time in SOCODIS system. The detailed design of the SOCODIS system has already been described in a previous study [4]. The actual and schematic diagram of SOCODIS system and reflective SODIS (SODIS_(Ref.)_) in this study is shown as Fig. 1 [25] while the transparent and absorptive SODIS (SODIS_(Abs.)_) cases are not presented. Both SODIS and SOCODIS system was exposed to direct sunlight on the rooftop of the same building where the rooftop RWH system was installed and it was located 37°27'18.31"N, 126°57'3.25"E. There was no need of any special permission to carry out the exposure experiments at the rooftop of the building and the activities did not involve endangered or protected species.

The duration of natural sunlight exposure in both systems, SODIS and SOCODIS was about 9 hours. Experiments were performed at all weathers in case of simple SODIS system but for SOCODIS system, the analysis was made only under mild and weak weather conditions. Sunlight radiations, at all occasions within a year, were monitored on-site with a SP-110 Pyranometer (Apogee Instruments Inc., Logan, USA) connected to a data logger (DT80 Series 2) recording 1 minute averages in Watt/m^2^ (W/m^2^). The spectral range of the Pyranometer used was 0–1750 W/m^2^. The values for irradiance and the temperature inside the bottle were recorded at an interval of an hour.

### Microbial analysis

The microbial water quality analysis was carried out by Standard Methods in Soil and Water Quality Laboratory, Seoul National University (SNU) at room temperature 25°C. The detection of *P. aeruginosa* was made by multiple tube method. Asparagine broth (Titan Biotech Ltd.) for the presumptive stage of *P. aeruginosa* was used in a series of fifteen test tubes for all three dilutions of 10, 1 and 0.1 ml and thus 5 tubes per each dilution. The tubes were than incubated at 35°C for 24 hours. To check the number of *P. aeruginosa*, the tubes were examined under black light after 24 and then after 48 hours. The tubes with green fluorescent pigments were selected and then 0.1 ml of culture was further incubated at 35°C into acetamide broth (Titan Biotech Ltd.) to complete the confirmation stage [26]. For *E. coli* detection and measurements, DifcoTM Lauryl Tryptose Broth (Becton, DickinsonandCompany) was used for the presumptive phase while the confirmation was done by using the BactoTM EC Medium with Mug (Becton Dickinson France S.A.).

### Miscellaneous measurements

A dark control was separately kept in 1 PET bottle wrapped in aluminium foil, over the same time period for each experiment. Nearly 5–6 repetitions were carried out for most of the analysis to avoid any experimental error and the error bars are shown in almost all the time graphs.

For the effects of different initial pH values, four pH values were chosen for both SODIS and SOCODIS system and the comparison was made only at weak weather conditions. For initial pH adjustment in stored rainwater, diluted HCl and NaOH were used. To check the effect on *P. aeruginosa* inactivation at different turbidity values, kaolin was introduced in raw rainwater samples. The turbidity values were set at 20NTU and 100 NTU and the effects were checked and compared for best suited case SODIS_(Abs.)_ and for SOCODIS system, separately.

The behavior of inactivation curves on experimental data was investigated with GInaFit (Geeraerd and Van Impe Inactivation Model Fitting Tool) using Geeraerd Log-linear plus shoulder model [27]. Geeraerd model has been widely used in many SODIS scientific contributions to fit experimental results [28].

## Results and Discussions

Table 1 shows the average initial values of physicochemical parameters (turbidity, pH, EC & DO) and the number of *P. aeruginosa* observed in stored rainwater samples. The physicochemical parameters were only used as reference values and the major emphasize of this study was on the microbial analysis. The concentration of *P. aeruginosa* in harvested rainwater before disinfection varied throughout the year as 350–900 CFU/100 ml depending upon both wet and dry seasons. No artificial method of *P. aeruginosa* or *E. coli* spiking was used.

**Table 1:** Reference values of stored rainwater samples.

Physicochemical parameters					Microbial parameter	
Turbidity	Temperature	pH	EC	DO	*P. aeruginosa*	*E. coli*
*NTU*	*°C*		*µs/cm*	*mg/l*	*CFU/100 ml*	*CFU/100 ml*
2–5	22–26	7–8	150–450	6–9	350–900	200–450

Chemical and microbiological parameters fluctuated during the course of the study, with the highest levels of microbiological contamination in wet seasons when the sunlight irradiance were highest and sunny weather conditions prevailed. Overall, the collected rainwater had a relatively good physicochemical quality but did not meet the requirements for potable water. Most of the water-quality parameters including the bacterial counts were higher both at the beginning and ending of the wet season mainly due to dirty catchment surfaces and addition of first-flush into rainwater tanks after long dry periods. Initial pH was high in dry periods but decreased during the rainy season and increased again after the rains stops. Both *E. coli* and *P. aeruginosa* levels were lower during the wet season due to short water storage time despite the fact that the fresh contamination was added with the rains. Due to filtration of the rainwater through the filters attached to the weirs before entering into the rainwater storage tank, a reduction in the concentrations of *P. aeruginosa* was expected, but this was not always the case. This was evident by observing the microbial load even after the filtrations step and by observing the good numbers of microorganisms even after the first-flush.

### Evaluation of solar radiation and temperature under different weather conditions

**Figure 2:**
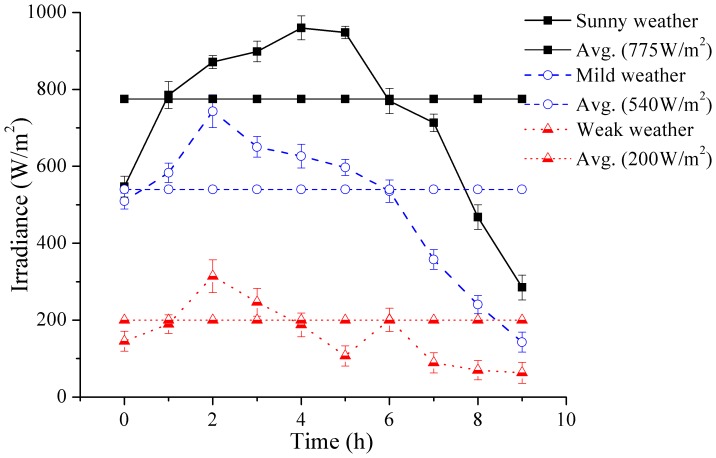
Irradiance change under different weather conditions.

Solar irradiance plays the most important role in solar based disinfection systems. For efficient SODIS, a geographical area should receive radiation above 500 W/m^2^ for 3–5 hours [19], [29]. Fig. 2 represents the pattern of sunlight intensity by using the hourly average values of irradiance for categorizing the weather condition into sunny, mild and weak weathers. The time 0h corresponds to 8am or 9am in the morning while 9h corresponds to 5pm or 6 pm in the evening. The irradiance values varied from 700–950 W/m^2^ for sunny weather with an average of 775 W/m^2^ (usually from May to August), 500–700 W/m^2^ for mild weather with an average of 540 W/m^2^ (usually from September to October and March to April) and 140–300 W/m^2^ with an average of 200 W/m^2^ for weak weather (usually from November to February).

**Figure 3:**
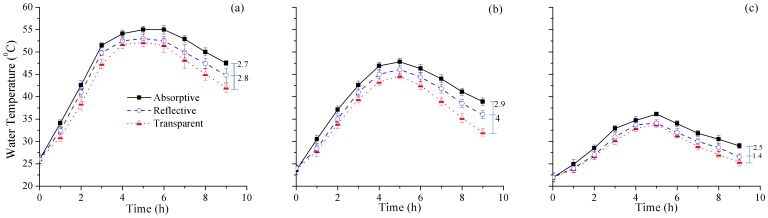
Temperature change in PET bottles with different rear surfaces against exposure time under (a) sunny, (b) mild, and (c) weak weather conditions.

The temperature difference at 9th hours between absorptive and reflective rear surfaces container was 2.5–3°C under all weather conditions. Temperature comparison was also made using the same rear surfaces as mentioned earlier under different weather conditions. The water temperature was measured at regular intervals of 1h by keeping the device inside the bottles while the ambient temperature was not recorded. The average temperature values drawn for sunny, mild and weak weather conditions are shown in Fig. 3(a, b, & c). Absorptive container used the mechanism to enhance the water temperature by absorbing radiation while the irradiance affect was dominant in reflective container.

There was almost an exponential rise in water temperature reaching maximum value in the afternoon after which it drops with decreasing sun irradiance. Temperature was affected by weather conditions and was directly proportional to irradiance. This affect can be affirmed by comparing Fig. 2 with Fig. 3. The effective temperature for microbial inactivation was considered above 50°C with dominant thermal effect of sunlight [30]. The results of the current study were comparable with the previous findings as the temperature was raised above 50°C, during sunny weather conditions, in almost all containers shown in Fig. 3(a). However, mild weather conditions may not be fit for SODIS if only thermal rather than synergistic effects of thermal and UV radiations are considered since the maximum temperature attained was less than 50°C. Low temperature under weak sunlight conditions may have adverse effects on the efficiency of SODIS because of less or no thermal and/or synergistic effects of thermal and UV radiations. The observed values of temperature and irradiance were according to the weather pattern of South Korea (Latitude: 37°35’ North, Longitude: 127°03’ East). Similar pattern of temperature and irradiance can be observed for exposure time and can be generalized for any location in the world.

### Effects of different rear surfaces of PET bottles under different weather conditions in SODIS

**Figure 4:**
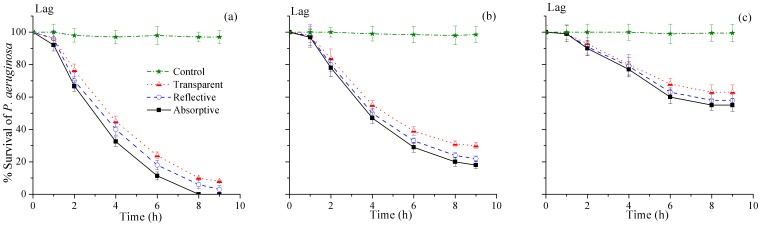
*P. aeruginosa* inactivation with different rear surfaces of PET bottles under (a) sunny, (b) mild, and (c) weak weather conditions.

The optimization of system by selecting the best type of container suited under different weather conditions was one of the main objectives of this study. For this purpose, transparent, absorptive and reflective containers were used to compare the efficacy of SODIS at sunny, mild and weak weather conditions as shown in Fig. 4 (a, b & c). Fig. 4(a) shows microbial inactivation in all three containers with different rear surfaces under sunny weather conditions. The complete inactivation of *P. aeruginosa* in absorptive container confirmed the dominant effect of thermal inactivation. Also the higher inactivation of *P. aeruginosa* (about 97%) in reflective container showed that synergistic effect of temperature and radiation was available. The inactivation rate under sunny weather condition with different rear surface was in order as absorptive>reflective>transparent containers. In transparent container the temperature was raised above 50°C under sunny weather condition but the complete inactivation of *P. aeruginosa* was not achieved even after 9h of exposure. This observation showed the resistivity of *P. aeruginosa* to SODIS and was contrary with the previous studies (22, 31, 32]. Under mild weather conditions as shown in Fig. 4(b) however, the inactivation rate of *P. aeruginosa* was unexpectedly higher in reflective containers compared to absorptive containers but the complete inactivation of *P. aeruginosa* was not achieved in any case. The exact reason of this slightly higher inactivation in reflective container was unknown at least in this study.

The difference in inactivation of *P. aeruginosa* with transparent rear surface under mild conditions was about 10%. As none of the water sample, with any rear surface, under mild weather condition could reach to the 50°C so the irradiance, other dominant factor under SODIS, may be the possible reason for *P. aeruginosa* inactivation. The effects of irradiance looked to be more significant with reflective rear surface as indicated in Fig. 4(b). Transparent containers showed poor performance where by both effects of radiation and temperature were not concentrated as was the case in reflective and/or absorptive containers. Weak weather as displayed in Fig. 4(c) showed bad results for similar effects of different rear surfaces. Only 37–45% inactivation of *P. aeruginosa* after 9 hours of exposure to direct sunlight showed that least effect of temperature and/or irradiance was available.

In all above mentioned conditions, except SODIS_(Abs.)_ under sunny weather condition, the inactivation rate of *P. aeruginosa* was almost negligible for first hour. This duration represents the lag phase in which the bacteria showed some resistance to solar inactivation. After lag phase, exponential inactivation of *P. aeruginosa* was observed for further 3–5 hours which was the peak hours of sunlight irradiation on any day and under all weather conditions. For last 2–3 hours of exposure, there was almost negligible inactivation at least under weak sunlight intensity.

### Comparison of SODIS_(Ref.)_ and SOCODIS systems for radiation and temperature effects on microbial inactivation

**Figure 5:**
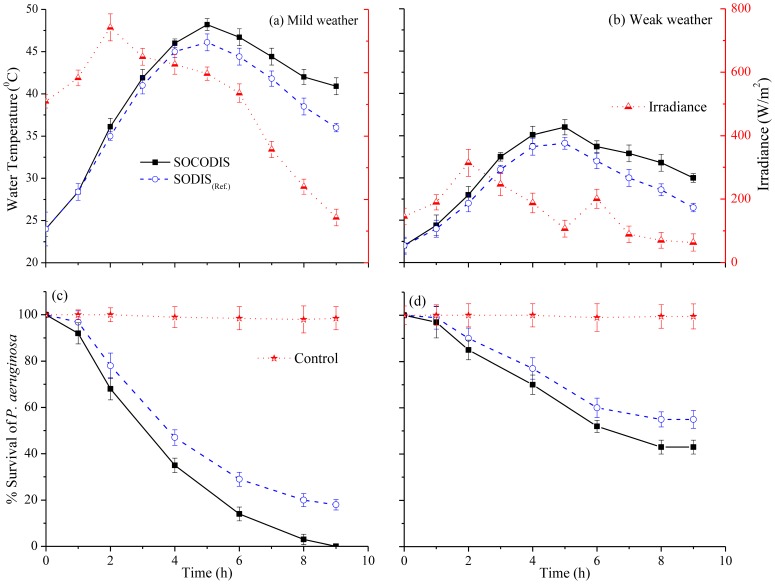
Comparison of temperature and *P. aeruginosa* inactivation in SODIS_(Ref.)_ and SOCODIS system under mild (a & c), and weak (b & d) weather conditions.

The efficiency of SODIS and SOCODIS systems was investigated under different weather conditions based upon the irradiance range and temperature. During 9 hours exposure to sunlight, the inactivation rate of *P. aeruginosa* was initially measured after 1h and then after every 2h interval. Finally the comparison of SODIS(_Ref.)_ was made with SOCODIS system. All the results exhibited almost the same trend observing a close correlation between irradiance and time required to inactivate *P. aeruginosa*. At first, for the comparison purposes of SODIS_(Ref.)_ and SOCODIS, the temperature changes under mild and weak weather conditions were measured at regular intervals of 1h. The average temperature values of all four bottles, kept inside the solar collector, for mild and weak weather conditions are shown in Figs. 5(a) and 5(b), respectively. Comparing the maximum temperature reached in SODIS and SOCODIS system, difference between peak values under mild and weak weather conditions was about 2–3°C. The relatively greater temperature increase in SOCODIS system was observed because the solar irradiation was reflected back onto the PET bottles from all four sides due to the open side wings of solar box. This effect was achieved only from one side i. e. base of the PET bottle, in SODIS _(Ref.)_ case. The synergistic effects of temperature and irradiance were also reported in earlier studies [31] which were evident under mild weather conditions as shown in Fig. 5(c) where the complete inactivation of *P. aeruginosa* was achieved.

However, the SOCODIS system remained inefficient under weak weather conditions, mostly because the synergistic effect of radiation and temperature was absent shown in Fig. 5(d). The difference in disinfection efficiency between the two systems can be summarized as follows: the SOCODIS system was about 20–30% more efficient than the simple SODIS system. It is well known previously that the effectiveness of SODIS improves when a solar collector is used, enhancing the irradiance effect by reflecting and returning the solar energy to the water samples, as support for bottles [31].

The exact mode of action by which SODIS or SOCODIS systems achieved *P. aeruginosa* inactivation is not fully understood. It has recently been demonstrated that solar photons attack proteins in bacteria, directly or indirectly via reactive oxygen species (ROS), causing protein oxidation. This leads to the disruption of metabolic function and damage to the cell membrane losing the bacterial cultivability [33]. *P. aeruginosa* inactivation is directly related to the sunlight intensity and UV dose, especially in the UV-A range. According to some previous studies, after getting the required UV dose, the disruption of bacterial cells continues even the exposed water samples are removed from sun and stored in dark conditions [33], [34].

UV-A is highest as a proportion of solar irradiance when the sun is at its highest altitude near solar noon, as is clear from an increase in the inactivation rate at this time, from 11 am to 3 pm in conducted experiments. All the results showed same tendency signifying a close relationship between UV dose and time required to inactivate microorganisms. One suspicion on solar disinfection by using PET bottles is the leaching of carcinogenic degradation agents from plastic bottles [35]. However, this suspicion was removed in another sunlight exposure study, PET degradation products such as terephthalate monomers and dimers are primarily formed at the outer surface of the bottles [36]. Some compounds including the carbonyls and plasticizers are found in the water but within the safe limits set for drinking water quality [37].

#### Effects of different initial concentrations of *P. aeruginosa* on disinfection efficiency

**Figure 6:**
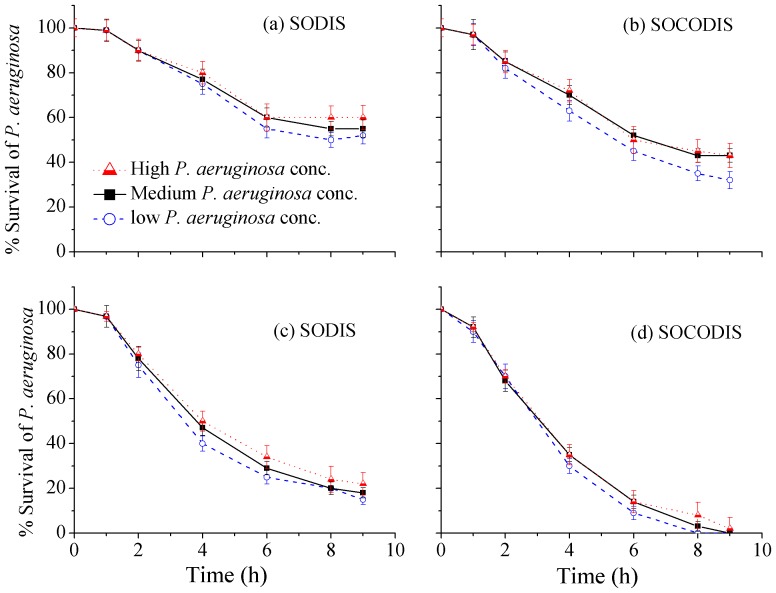
Effects of different initial *P. aeruginosa* concentratinos on disinfection efficiency in both SODIS and SOCODIS system under weak (a & b) and mild (c & d) weather conditions.

Since the initial concentrations of *P. aeruginosa* varied throughout the year depending upon the catchment conditions which in turn is influenced by the wet and dry seasons, therefore, the effects of this different initial concentrations on the disinfection efficiency was investigated at mild weather and the results are presented in Fig. 6 for both SODIS and SOCODIS system. Three different initial concentrations of *P. aeruginosa* (350, 700 and 980 CFU/ml) were chosen at different times during the four-months span of each weak and mild weathers but mainly at the beginning and ending of the both weather conditions.

Low to high concentrations of *P. aeruginosa* were possible under weak weather conditions due to both wet and dry season for four months from November to February. Mild weather conditions spanning nearly over four month (March, April, September and October) yielded medium to high concentrations of *P. aeruginosa* during March and April, due to relatively dirty catchment surface as a result of dry seasons, and low to medium concentrations, during relatively wet months and resulted clean catchment surface, in September and October. As shown in Fig. 6, there is very little effect of higher initial *P. aeruginosa* concentrations on disinfection efficiency especially in SODIS under both weak and mild weather conditions. The inactivation of *P. aeruginosa*, however, increased slightly about 5–10% by lowering the initial concentrations in SOCODIS system and this could be due to the concentrated effects of sunlight radiations.

#### Effects of the presence of *E. coli* on disinfection of *P. aeruginosa*

**Figure 7:**
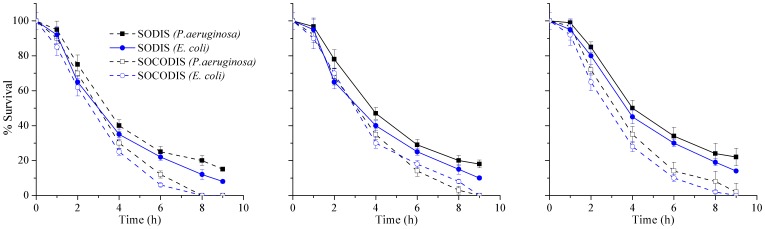
Effects on *P. aeruginosa* inactivation in both SODIS and SOCODIS system at mild weather at (a) low, (b) medium, and (c) high concentrations of *E. coli.*

The effects of the presence of other bacterial organisms like *E. coli* on disinfection efficiency of *P. aeruginosa* was investigated and for this purpose, three different initial concentrations of *E. coli* (210, 320 and 450 CFU/100 ml as low, medium and high, respectively) were chosen under mild weather conditions. It can be concluded from the results presented in Fig. 7 that the presence of other bacterium like *E. coli* had little effects on the disinfection performance of SODIS or SOCODIS system for *P. aeruginosa*. Higher *E. coli* concentrations, however, slightly decreased the inactivation of *P. aeruginosa* in addition to longer lag period, as is shown in Fig. 7 and this might be due to the increasing competition of different organisms for absorbing sunlight radiations or due to the bacterium selectivity of the UV-A radiations especially during early hours of exposure.

### Reaction kinetics

Inactivation kinetics of *P. aeruginosa* was investigated by using the Geeraerd Log linear and shoulder model in GInaFit and inactivation rate constant (*k*_max_) in all cases of SODIS and SOCODIS systems was calculated and compared. The model was originally defined by coupling two differential equations [27].
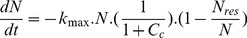
(1)

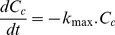
(2)


Where C_c_ is related to the physiological state of the cells, *k*_max_ is the specific inactivation rate [1/time unit] and N_res_ is the residual population density [CFU/mL]. Following format of the model is used when actually applying to the experimental data in GInaFit.
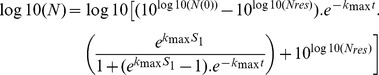
(3)


The first two mentioned equations (eq.1&2) can be obtained by substituting S_L,_ a parameter represents the shoulder (time unit), or N_res_ equal to zero. Table 2 presents a brief overview of *P. aeruginosa* inactivation based on the inactivation rate constants (*k_max_*), calculated using the Geeraerd model.

**Table 2:** Comparison of *k_max_* (1/min) for *P. aeruginosa* disinfection between SODIS and SOCODIS system under different weather conditions (Rainwater with low turbidity (2-5NTU) and neutral pH values).

Weather conditions	SODIS			SOCODIS
	Transparent	Absorptive	Reflective	
Sunny	0.47	0.63	0.56	ND*
Mild	0.17	0.22	0.26	0.71
Weak	0.07	0.1	0.11	0.26

* Not determined.

The *k_max_* (1/min) values in Tables 2 and 3 were calculated based on the Geeraerd model, as described earlier, and represent the inactivation rate constants for *P. aeruginosa*, with a corresponding coefficient of determination (R^2^) of 1, except where mentioned. A complete application of the model, i.e. with tailing and shoulder, was not observed in most of the cases and modeling with tailing was unlikely for the observed data probably due to the synergistic effects of UV radiations and temperature. For mild and weak weather conditions, temperature less than 50°C but high *k_max_* values validated the assumption that irradiance effects were significant in case of SOCODIS unlike SODIS for the inactivation of *P. aeruginosa*. This finding is in compliance with previously reported results [38].

**Table 3:** Comparison of *k*_max_ (1/min) for *P. aeruginosa* disinfection between SODIS_(Abs.)_ (suny weather) and SOCODIS system (mild weather) at different pH and turbidity values.

pH (under Turbidity∼5NTU)	Sunny weather	Mild weather	Turbidity (under pH∼7)	Sunny weather	Mild weather
	SODIS_(Abs.)_	SOCODIS	NTU	SODIS_(Abs.)_	SOCODIS
3	0.83	0.98	∼5	0.63	0.71
5	0.72	0.83	20	0.48	0.55
7	0.63	0.71	50	0.39	0.36
10	0.59	0.62	100	0.28	0.22

### Effects of different pH and turbidity values on disinfection efficiency

**Figure 8:**
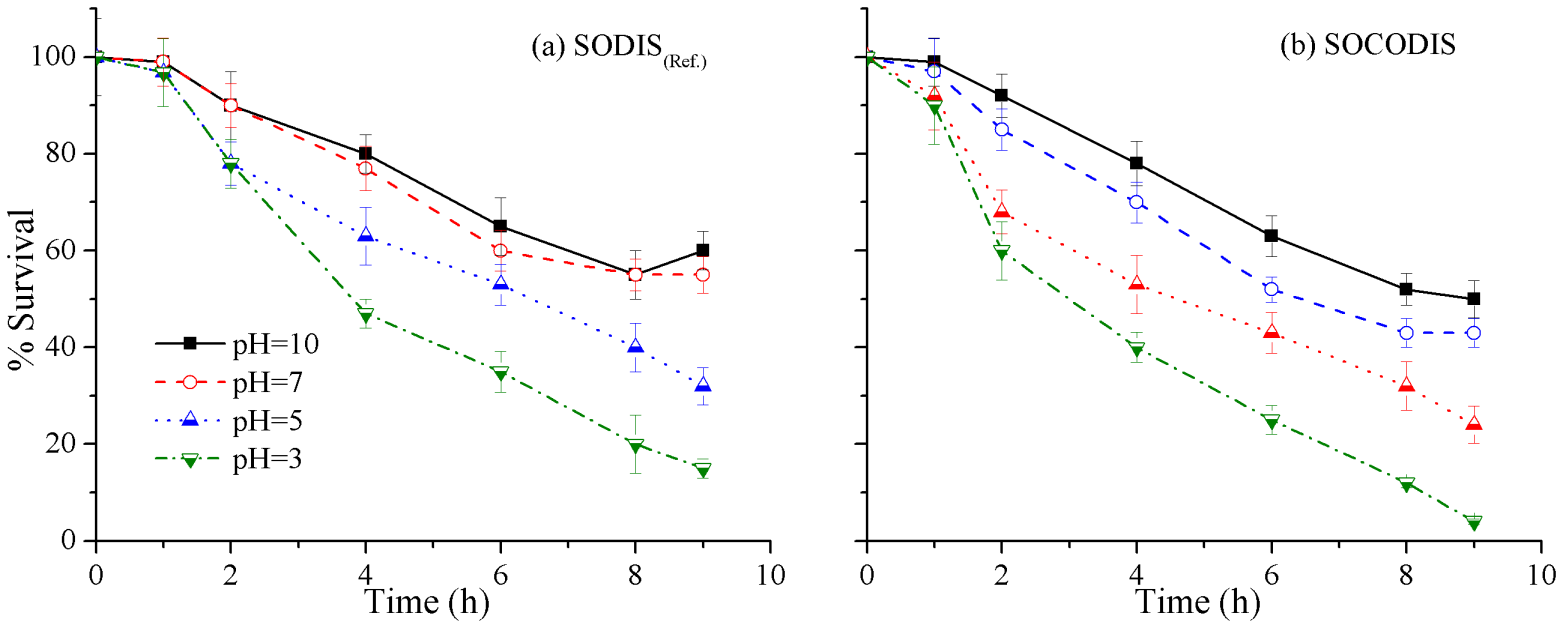
Effects of different initial pH values on *P. aeruginosa* inactivation in (a) SODIS_(Ref.)_, and (b) SOCODIS system at weak weather conditions.

**Figure 9:**
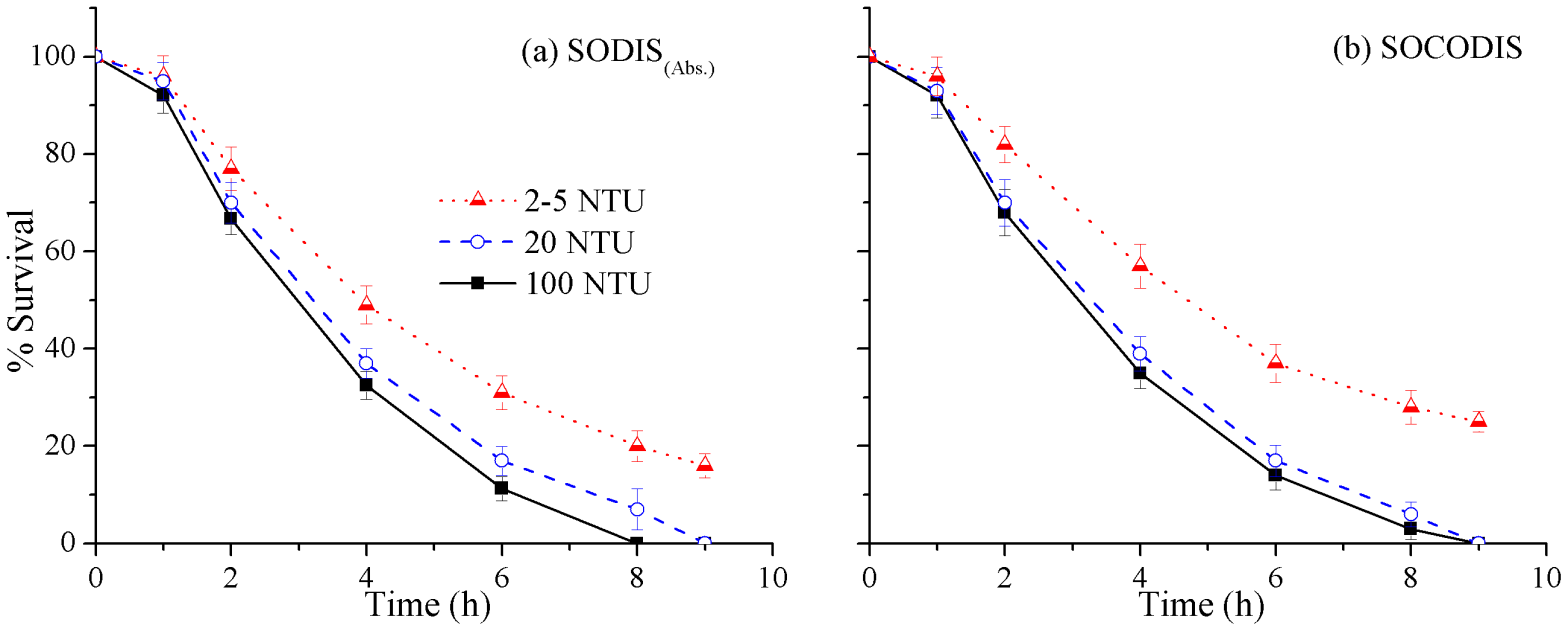
Effects of different turbidity values on *P. aeruginosa* inactivation in (a) SODIS_(Abs.)_ (sunny weather), and (b) SOCODIS system (mild weather).

Both pH and turbidity of the stored rainwater are among the main factors that may change the efficacy of solar disinfection systems. So, the effects of different initial pH and turbidity values on the efficiency of the solar disinfection systems were tested and the results are presented in Figs. 8 and 9, respectively. As mentioned earlier, diluted HCl and NaOH were used for adjusting initial pH values while kaolin was added for different initial turbidity values. As shown in Fig. 8, four initial pH values i.e. 10, 7, 5 and 3 were selected with rainwater with a low initial turbidity value (<5 NTU) and the comparison was performed only under weak weather conditions mainly due to inefficiency of the SOCODIS system at this weather. The overall disinfection efficiency increased by about 15% by lowering the initial pH value from 10 to 3 and nearly complete inactivation of *P. aeruginosa* was achieved at the acidic conditions. Low pH may have increased the inactivation of *P. aeruginosa* by presenting significant additional stress to the cells [5], [39].

Table 3 shows a comparison of the *P. aeruginosa* inactivation between SODIS and SOCODIS system under mild and sunny weather conditions at different initial pH values. These findings differed from the earlier studies stating no effects of pH values on microbial inactivation rates [40]. In this study lowering initial pH values had significant effect of *P. aeruginosa* inactivation as reported previously [41]. It is worth mentioning that there are no health-based guidelines for pH and it is reported that the consumption of food or beverages with low (2.5) or high (11) pH does not result in adverse health effects [42].

For investigating the effects of initial turbidity values with rainwater having nearly neutral pH values, best suited solar disinfection cases, SODIS(_Abs.)_ under sunny weather and SOCODIS system under mild weather condition were selected for comparison as shown in Figs. 9(a) and 9(b), respectively. Rainwater samples collected from an underground storage tank had a turbidity value of less than 5 NTU. For comparison purposes, higher values (20 and 100NTU) were achieved by adding kaolin. Samples with low turbidity showed better results; the difference between inactivation of *P. aeruginosa* was insignificant up to about 20 NTU. However, the SOCODIS system showed poor performance in terms of *P. aeruginosa* inactivation at higher turbidity, and disinfection efficacy decreased by almost 10–15%. This could be because of a loss of UV radiations due to scattering and absorption by suspended particles in rainwater samples. Various microbiological studies have revealed that highly turbid waters do not allow the synergistic effect to occur [22], [31], [38], [43]. However, the highly turbid water may undergo a substantial temperature increase because of the absorption of solar radiation by suspended particles, which may contribute to higher disinfection efficiencies [22]. The case of SODIS(_Abs.)_ system under sunny weather conditions was in accordance with previously reported literature but contrarily the complete inactivation of *P. aeruginosa* was not achieved.

Table 3 summarizes the change in the inactivation rate constants of *P. aeruginosa* with the variable values of turbidity when Geeraerd model was used for best suited cases of SODIS(_Abs.)_ and SOCODIS systems. As can be noted from the Table, the values of *k*_max_ were decreased with the increased turbidity level in all cases. Surprisingly, however, the difference between the *k*_max_ values for turbidity level 20 and 100 under SODIS_(Abs.)_ was lesser (0.20) as compared with SOCODIS case (0.33). It can be due to the substantial temperature increase because of the absorption of solar irradiance by suspended particles [22] when the rear surfaces of PET bottles were painted black.

## Conclusion

SODIS_(Abs.)_ under, sunny weather conditions, was the most efficient case (100% inactivation) as compared to the SODIS_(Trans.)_ (92%) and SODIS_(Ref.)_ (97%) cases for the inactivation of *P. aeruginosa* in harvested rainwater. This could be due to the thermal and/or synergistic effect of temperature and irradiation. Generally, a 20% increase in the disinfection efficiency of the SOCODIS system, even under mild weather conditions, suggested that the simple solar collector/box is an efficient, cost effective and user friendly option especially for remote areas. However under weak weather conditions, a longer exposure time or other supplementary means with the SOCODIS system are required for the complete inactivation of *P. aeruginosa*.

There was very little effect of high initial *P. aeruginosa* or *E. coli* concentrations on disinfection efficiency but the inactivation of *P. aeruginosa* increased slightly about 5–10% by lowering the initial concentrations in SOCODIS system. Higher *E. coli* concentrations, on the other hand, slightly decreased the inactivation of *P. aeruginosa* in addition to longer lag period. Although, not completely inactivated under weak weather conditions, the overall disinfection efficiency increased by about 15% by lowering the initial pH value from 10 to 3 especially in SOCODIS system. Above 20 up to 100NTU, 16–25% decrease in activation of *P. aeruginosa* suggested that water should be filtered before applying solar disinfection. Overall, the inactivation of *P. aeruginosa* in harvested rainwater by solar disinfection systems can be considered a useful addition in making the stored rainwater available for potable purposes. The detailed investigation and disinfection of *P. aeruginosa* with sunlight based disinfection system under different weather conditions and variable parameters will help researchers to understand and further improve the innovative SOCODIS system. This can also be viewed as an easy approach for the poor communities living in the developing world in accessing the potable water using efficient and cost-effective point-of-use treatment methods.
